# Health monitoring during water scarcity in Mayotte, France, 2017

**DOI:** 10.1186/s12889-019-6613-8

**Published:** 2019-03-12

**Authors:** Marion Subiros, Elise Brottet, Jean-Louis Solet, Armelle LeGuen, Laurent Filleul

**Affiliations:** 1 Santé publique France, cellule d’intervention en région océan Indien (CIRE OI) [French public health agency in the Indian Ocean Region], Rue Mariaze, BP 410, 97600 Mamoudzou, Mayotte France; 2 Santé publique France, cellule d’intervention en région océan Indien (CIRE OI) [French public health agency in the Indian Ocean Region], 2 bis avenue Georges Brassens CS 61002 – 97743 Saint-Denis cedex 9, Saint-Denis, La Réunion France; 3 Agence de santé océan Indien [Indian Ocean Health Agency], Rue Mariaze, BP 410, 97600 Mamoudzou, Mayotte France

**Keywords:** Epidemiological surveillance, Epidemic, Water scarcity, Mayotte, Indian Ocean

## Abstract

**Background:**

During the 2016–2017 austral summer, unprecedented water scarcity was observed in the south of Mayotte, French island in the Indian Ocean. Therefore, authorities introduced restrictive measures to save the water of this part of the island. The rationing system affected over 65,000 people, for four months. In order to detect a possible deterioration of the health situation, a strengthened epidemiological surveillance system was set up.

**Methods:**

Surveillance focused on intestinal and skin diseases, which are often associated with a lack of hygiene or poor-quality drinking and bathing water. Three pathologies were monitored: acute diarrhoea, acute gastroenteritis and skin diseases and also, proportion of antidiarrhoeal and rehydration solutions sales in pharmacies. Cases of leptospirosis were also under surveillance. The analyses consisted of comparing the collected data according to the areas that were either affected or not affected by the water restrictions. Comparisons with historical data were also made.

**Results:**

Although none of the surveillance systems were able to demonstrate any impact on skin diseases, they revealed a very sharp increase in the proportion of consultations for acute diarrhoea and gastro-enteritis in the southern area. This was corroborated by a high increase in the sales of antidiarrhoeals and oral rehydration solutions via the sentinel pharmacists in the south of the island compared with those of the north. Comparison with historical data highlighted the occurrence of an unusual situation.

**Conclusion:**

These water restrictions caused a real deterioration in the health status of the inhabitants who were deprived of water.

## Background

Mayotte is a French island in the Comoros archipelago in the South-West Indian Ocean. It is 300 km away from the coasts of Madagascar and 400 km away from the coasts of Mozambique [1].

In January 2016, Mayotte’s population was estimated at 235,132 inhabitants. It is the youngest French department with a steadily growing population due to its high birth rate and high level of immigration [2]. In Mayotte, socio-economic conditions are generally poor. Indeed, 84% of the population is living beneath the metropolitan poverty threshold [3]. Living conditions remain precarious, with 37% living in shanties, 28% in housing with no access to running water and two thirds in housing without basic sanitation) [4].

The island of Mayotte has a maritime tropical climate with two seasons interspersed. The hot and humid season, from December to March (hot temperature: 24–32 °C, high humidity: 70–95%) includes tropical depressions, and more rarely, cyclones. This season provides 80% of the annual rainfall. The temperate and dry season or southern winter, takes place from June to September (temperature: 20–28 °C, 61–90% humidity) [1].

Surface resources from river surface waters and two hillside reservoirs represent 80% of all the drinking water that is produced [5]. Dzoumogne hillside reservoir feeds the north area while Combani reservoir feeds the center/south area. As a result, any delay in the rainy season’s arrival to replenish these resources causes water scarcity in the department.

**Fig. 1:**
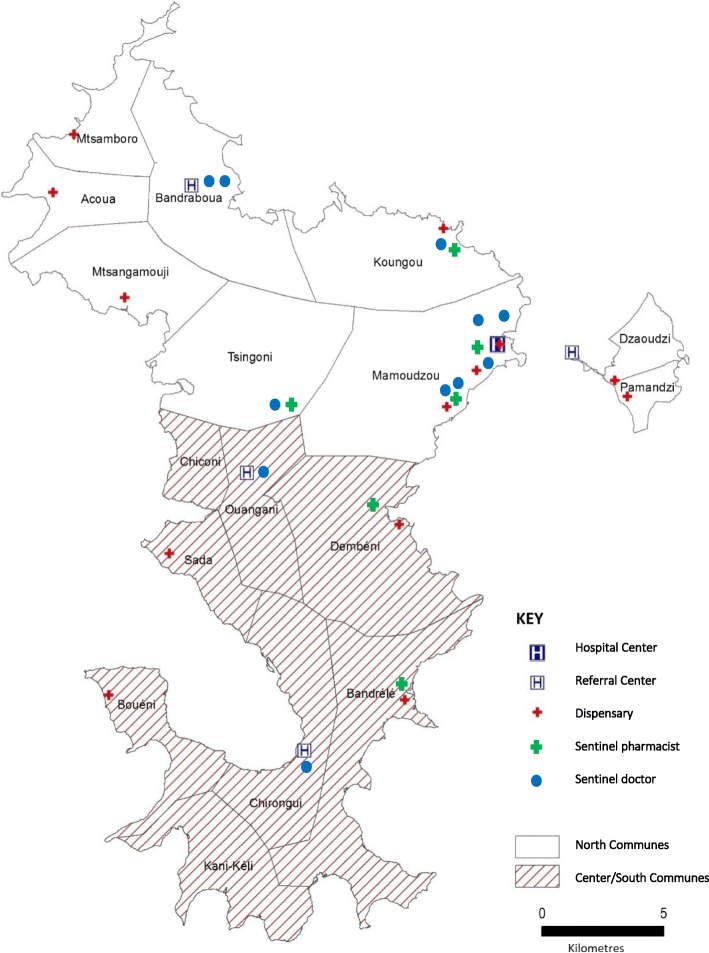
Map of the communes affected by the water restrictions (center/south and north), the Mayotte Hospital Center health centers, the sentinel pharmacists and doctors, 2016–2017 [Source: Geofla IGN, Produced by CIRE OI, 2017]

During the 2016–2017 austral summer, unprecedented water scarcity was observed in the center and the south of the island of Mayotte due to a lack of rain. The rainy season was considerably delayed as it did not arrive before March, eventually connected with global climate changes [6]. Consequently, in December 2016, the Combani reservoir was completely drained and in the absence of any other water supply solution, the prefecture of Mayotte decided to introduce water rationing (water restrictions) in the island’s eight central and southern communes: Bandrele, Dembeni, Chiconi, Sada, Ouangani, Chirongui, Boueni, Kani-Keli (Fig. 1). The inhabitants could only access tap water every two days between the 15th of December 2016 and 5th of April 2017. In January and February, the system applied its toughest restriction, allowing the access to water every three days only. As a result, the rationing system affected over 65,000 people (Institut national de la statistique et des études économiques, 2012), i.e. 31% of the population, during four months.

A disruption in the water supply exposes the population to major health risks [7] owing to the use of contaminated surface water for food and hygiene, the inability to evacuate excreta, and storage of water in tanks likely to contain mosquito larvae that are vectors of arboviruses. Moreover, water shortage is likely to lead to epidemic outbreaks of endemic diseases in Mayotte such as acute gastroenteritis, typhoid fever, hepatitis A, and leptospirosis [8, 9].

In order to detect a possible deterioration in the island’s health situation, a strengthened epidemiological surveillance system was set up by the French public health agency in the Indian Ocean region (CIRE OI). The CIRE OI had to take into consideration the distinctive features of Mayotte to optimise the quality and representativeness of the data collected.

The CIRE OI had been relocated to Mayotte just a few weeks before the water crisis started, on December 1st, 2016. Sentinel networks were previously managed remotely by the epidemiologists of the Reunion Island. The sentinel doctor’s network was fragile with eleven doctors, only two of whom were active in the center/south area. Meanwhile, the surveillance activity could be irregular and the new branch of the CIRE OI had the mission to develop and consolidate this network despite the difficulties related to the health care provision. Indeed, in Mayotte, the care offer is mainly provided through the Mayotte Hospital Center (CHM) of Mamoudzou and its 4 referral centers (CRs) and 13 dispensaries (Fig. 1). The private sector is very little developed with, in November 2017, only 22 private general practitioners being active throughout the territory. For comparison, among all sectors combined, there are 61 general practitioners and 40 specialist doctors per 100,000 inhabitants in Mayotte, compared to 142 general practitioners and 146 specialists on the island of Reunion, a neighbouring French department in the Indian Ocean [10]. Moreover, the turnover of health professionals in both sectors is extremely high and weakens the health system. It is hard to mobilize and involve the medical community in long-term surveillance projects.

## Methods

The objective of this surveillance was to monitor the health situation through the monitoring of indicators derived from health data (medical consultations, biological analyses, drug use). The ultimate goal of this monitoring was to provide reliable indicators to local authorities to adapt the management measures introduced in the context of the water crisis.

In parallel with the existing surveillance networks, the CIRE OI decided to set up a specific epidemiological monitoring thanks to volunteer doctors to ensure data collection throughout the period of the water crisis. The objective was to collect consultation data despite time and logistical difficulties: the CRs of the CHM were not computerized and the collection had to be done on paper via a questionnaire developed with the doctors. The transmission of the surveillance forms had to be as flexible as possible to avoid a waste of time to health professionals already overworked.

The surveillance system was introduced throughout the whole of the water crisis. It focused on intestinal and skin diseases, which are often associated with a lack of hygiene or poor-quality drinking and bathing water. Three pathologies were therefore monitored: acute diarrhoea (more than three liquid stools a day, occurring less than two weeks prior to the consultation), acute gastroenteritis (fever, diarrhoea and nausea/vomiting), skin diseases (abscesses, fungal infections and impetigo). Drugs’ sales associated to acute diarrhoea and gastroenteritis were also monitored. Leptospirosis cases were monitored thanks to CHM’s laboratory data. The aggregated data collected were not personal data and no patient information was analysed.

Descriptive analyses were conducted. Comparative statistical analyses depended on data from each surveillance network (availability of historical data in particular). Thus, the indicators studied were compared according to the geographical area (impacted or not by the water restrictions) or the monitoring year (normal year versus year of the water crisis). Comparisons between the different independent groups were made using the Chi2 test. When establishing comparative statistics, a value of *p* < 0.05 was considered statistically significant. The analyses were performed with Stata12.

The surveillance incorporated several systems and analyses elaborated in Table 1.

**Table 1:** Strengthened epidemiological surveillance system during the water scarcity period in Mayotte, 2016–2017

Surveillance system	Surveillance Indicators	Data transmission to CIRE OI	Statistical analyses
Epidemiological surveillance in the CHM referral centers (CRs)	• Weekly percentage of consultations for acute diarrhoea and gastroenteritis (number of consultations for theses pathologies compared to total number of weekly consultations) • Weekly percentage of consultations for skin diseases (number of consultations for skin diseases compared to total number of weekly consultations)	Data sent every day or every week by each doctor by phone, fax, scan, courier or a pickup directly on site by CIRE OI team	Kahani and Mramadoudou CR’s data (center/south area) compared to Dzoumogne CR’s data (north area)
Mayotte network of sentinel doctors	• Weekly percentage of consultations for acute diarrhoea (number of consultations for acute diarrhoea compared to total number of weekly consultations)	Data sent weekly via a form online, accessible with username and password by each doctor	- Kahani and Mramadoudou doctor’s data (center/south area) compared to nine other doctors’ data located in the non-impacted area, during water crisis - Comparison of central/south doctors’ data during crisis and in a normal year
Mayotte network of sentinel pharmacists	• Weekly percentage of antidiarrhoeal drugs: Smecta®, Tiorfan® and rehydration solutions (number of sales for these drugs compared to weekly total number of patients)	Data sent weekly by email in an Excel database, by each pharmacist	- Bandrele and Dembeni pharmacists’ data (center/south area) compared to four other pharmacist’s data located in the non-impacted area, during water crisis - Comparison of central/south pharmacists’ data during crisis and in a normal year
Epidemiological surveillance with the CHM’s laboratory	• Number of leptospirosis cases confirmed at the CHM’s laboratory	For each new confirmed case, data sent by email by the CHM’s laboratory to ARSOI and CIRE OI	Descriptive analysis according to the residence village of the patient, impacted or not by water restrictions and comparison with data since 2014.

**Specific epidemiological monitoring** was introduced from the first day of the water restrictions in two of CHM’s referral centers (CRs) likely to receive patients exposed to the restrictions (Mramadoudou in the south and Kahani in the center) and in one unexposed CR (Dzoumogne in the north). Data were collected daily using a set collection form by each volunteer doctor from the CRs and weekly forwarded to the CIRE OI. The analyses consisted in comparing the number of consultations for digestive and skin symptoms in the two exposed centers with the unexposed comparator center. This surveillance spanned from 15th of December 2016 to 15th of April 2017.

**The network of sentinel doctors**, set up in 2009, is based on voluntary doctor participation (consulting in hospitals or private practice). The network’s aim is to analyse the seasonality and epidemiology of pathologies such as flu-like syndromes, acute diarrhoea, dengue-like syndromes and bronchiolitis. Each week, the sentinel doctors report the total number of weekly consultations and the number of consultations for each of the four pathologies. For this study, it focused on the results for acute diarrhoea from 15th December 2018 to 15th April December and historical data are also presented. During the period of water scarcity, eleven sentinel doctors were registered on the network (including one private practice doctor) and two of them worked in the center/south area affected by the restrictions (Mramadoudou and Kahani).

**The network of sentinel pharmacists** aims to detect an unusual health situation by monitoring the distribution of certain drugs of interest like certain antipyretics and antidiarrhoeals. It relies on the voluntary participation of six private pharmacies spread across the island (two of which were in the center/south area). Each week, these pharmacies fax or email a statement of the number of boxes sold during the previous week in order to monitor acute gastroenteritis (Smecta®, Tiorfan® and oral rehydration solutions (ORS)). The total number of patients who visited the pharmacy in the previous week was also recorded to obtain a denominator to calculate a weekly sales percentage for each molecule monitored. For this study, it focused on the monitoring for acute gastroenteritis from 15th December 2018 to 15th April December and historical data are also presented.

**Epidemiological surveillance of leptospirosis** with the CHM’s laboratory has been in place in Mayotte since 2008. All cases biologically confirmed by the CHM’s laboratory are reported to the Indian Ocean Health Agency (Agence de Santé océan Indien: ARS OI) in Mayotte [11]. Each confirmed cases were investigated and collected data are regularly analysed by the CIRE OI. The geographic information is the residence village of the patient. The descriptive analysis presents data for the epidemic period in Mayotte: from January to March. Data for 2017 are compared to historical data (2014 to 2016).

Finally, as part of its surveillance and health alert missions, the CIRE OI remained the recipient of any event that could have an impact on public health. These reports can come from any partner of the health surveillance (health professionals especially).

The CIRE OI coordinated and animated the surveillance networks. Every day, health professionals were made aware of the need for early and reactive data collection and transmission. On a daily basis, the CIRE OI entered and analysed data collected in various databases. A weekly analysis allowed the production of epidemiological points for weekly feedback sent to data providers by email or during staff meeting. Lastly, a health situation overview was presented weekly to the local authorities (including the ARS OI) to help them in setting up or adapting measures to manage the potential epidemic risk.

## Results

Between December 2016 and April 2017, the CIRE OI produced two briefing notes and ten epidemiological feedback points towards its partners and the health authorities in order to alert them of the population’s changing health status.

### Surveillance of cases of acute diarrhoea and gastroenteritis

**Fig. 2:**
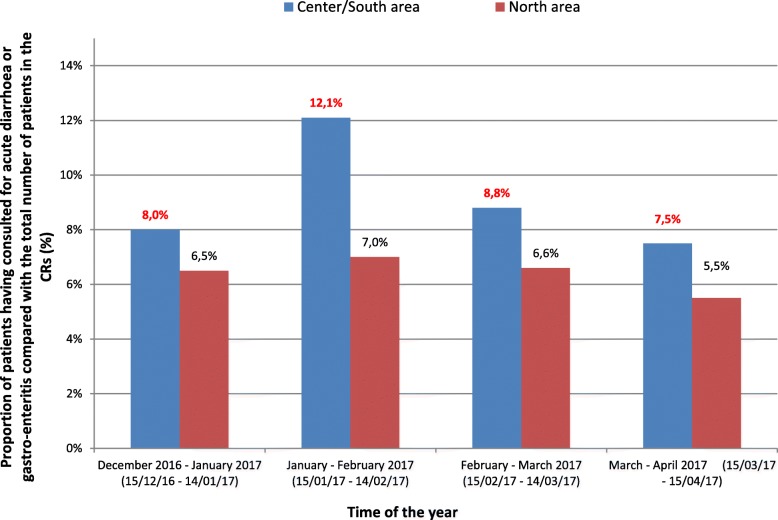
Monthly proportion of patients having consulted for acute diarrhoea or gastro-enteritis compared with the total number of patients in the CRs, according to the area impacted or not impacted by the water restrictions Mayotte, 15 December 2016 to 15 April 2017*. * Kahani and Mramadoudou CRs (Center/South) and Dzoumogne CR (North)

Epidemiological surveillance in the CHM’s CRs revealed a very sharp increase in the percentage of consultations for acute diarrhoea and gastro-enteritis in the center/south area. For the entire surveillance period, the percentage of consultations for acute diarrhoea and gastroenteritis was significantly higher (*p* < 10^− 4^) in the area impacted by water restrictions (9.4% i.e. 1053 out of a total of 11,172 consultations) compared to the non-impacted area (6.7% i.e. 302 out of a total of 4519). During the second month of surveillance, which followed tightened water restrictions, the percentage of consultations for acute diarrhoea and gastro-enteritis in the CRs in the center/south rose to 12.1% (i.e. 413 out of a total of 3421 consultations), compared to 7.9% during the first month of surveillance (i.e. 293 out of a total of 3678 consultations). This percentage then returned to the level observed in December 2016 from the first week of March. The percentage of consultations for acute diarrhoea and gastro-enteritis in the north of the island remained stable (around 6.3%, i.e. 344 out of a total of 5432 consultations between 15/12/2106 and 15/04/2017) during the crisis (Fig. 2).

**Fig. 3:**
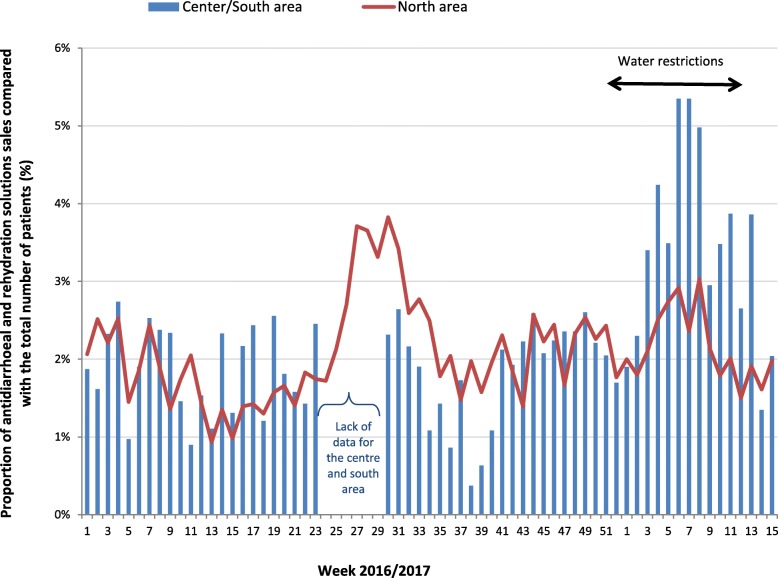
Weekly proportion of antidiarrhoeal and rehydration solutions sales compared with the total number of patients, according to the area impacted or not impacted by the water restrictions, sentinel pharmacists network, Mayotte, 2016–2017. * Bandrele and Dembeni pharmacies (Center/South)

The increase in the number of cases of acute diarrhoea and gastro-enteritis observed in the CRs in January and February 2017 was corroborated by a high increase in sales of antidiarrhoeals and oral rehydration solutions in the two sentinel pharmacists in the center/south area compared with those of the north (Fig. 3). These sales represented up to 5.3% (122 boxes sold for 2280 patients) for two consecutive weeks (W06–2017 and W07–2017) compared to 2.6% (195 boxes sold for 7465 patients) in the sentinel pharmacists not located in an area affected by the water restrictions.

During the entire surveillance period, the percentage of sales was significantly higher (*p* < 10^− 4^) in the center/south pharmacies (3.3% i.e. 579 out of a total of 17,653 consultations) compared to the other sentinels in the north (2.2% i.e. 1338 out of a total of 61,525). Besides, these data from those two center/south sentinel pharmacies were compared with historical data at the same period (from S50–2015 to S15–2016): a significantly higher percentage of sales was observed (p < 10^− 4^) in the year of water restrictions compared to previous years (1.9% i.e. 336 out of a total of 17,415 sales).

The data from the network of sentinel doctors showed an increase in acute diarrhoea cases above the seasonal averages between 30 January and 19 March 2017. This increase was largely influenced by the consultation data of the two sentinel doctors working in the center/south area. A peak in activity for acute diarrhoea was observed in week 07–2017, representing 67% of the sentinel doctors’ overall activity (87 consultations for acute diarrhoea out of a total of 129 consultations), i.e. a level never observed at this time of year since the network’s creation in 2009.

During the entire surveillance period, the percentage of consultations for acute diarrhoea was significantly higher (*p* < 10^− 4^) in the center/south area affected by water restrictions (10.1% i.e. 293 out of a total of 2895 consultations) compared to the non-impacted area (5.7% i.e. 105 out of a total of 1844). Moreover, these data from those two center/south sentinel doctors were compared with historical data at the same period (from S50–2014 to S15–2015): a significantly higher percentage (*p* < 10^− 4^) was observed in the year of water restrictions compared to previous years (3.8% i.e. 106 out of a total of 2803 consultations).

### Surveillance of cases of skin diseases

Regarding skin diseases (impetigo, abscesses, skin fungal infections), the surveillance were not able to demonstrate any impact caused by the water restrictions: the same proportion of consultation for these pathologies was observed across the entire territory, representing 10 to 13% of all consultations at the CHM’s CRs. No historical data were available to compare the situation with a normal season.

### Surveillance of cases of leptospirosis

**Fig. 4:**
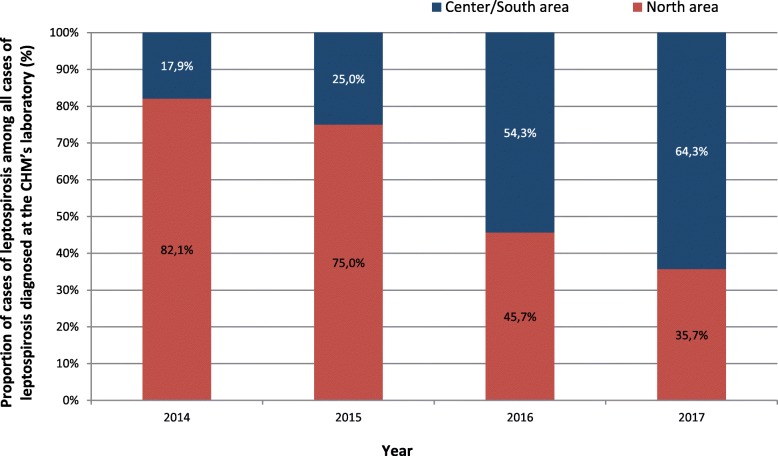
Proportion of cases of leptospirosis among all cases of leptospirosis diagnosed at the CHM’s laboratory (W01 to W13) according to the area impacted or not impacted by the water restrictions, Mayotte, 2014–2017

The water crisis struck Mayotte right in the middle of the leptospirosis epidemic season. The island is an endemic territory for this pathology where a surge of cases is always observed during the rainy season from January to March, with a mean annual incidence of 47 cases per 100,00 inhabitants [11]. In the first quarter of 2017, the number of cases of leptospirosis was higher in the communes impacted by the water restrictions compared with the northern communes: 64.3% in the center/south vs. 35.7% in the north. Center/south data represented 36 out of 56 cases confirmed by the CHM between January and March and we observed a significant difference (*p* = 0,01) comparing with historical data of 2014 epidemic period (5 out of 28 cases confirmed). The geographical distribution of cases occurring in from 2014 to 2017 is presented in Fig. 4.

## Discussion

During the austral summer, from December 2016 to April 2017, the population of Mayotte experienced an unprecedented water crisis. The delayed rainy season caused a water shortage that affected the island’s central and southern populations who were consequently subjected to water rationing for four months. This situation caused a real deterioration in the health status of the inhabitants deprived of water.

The epidemiological monitoring system revealed an increase in the number of medical consultations for acute diarrhoea and gastro-enteritis in the areas suffering these restrictions, corroborated by a sharp increase in the sales of antidiarrhoeals and oral rehydration solutions in the community pharmacies of these areas. In light of this observation, the local authorities then relaxed the restrictive measures, increasing access to running water to every two days (against every three days) during the last weeks of the crisis. While the link between heavy rainfall and an increased risk of diarrhoea is well established, less is known about the effect of drought on these epidemics. Some studies have identified the use of alternative water sources like ponds, wells and domestic water reservoirs (a very widespread practice in Mayotte) as the most immediate cause of acute diarrhoea epidemic outbreaks [12, 13]. A study conducted in Laos has also shown that water scarcity in the region of Luang Prabang triggers peaks of diarrhoea during the dry and hot season and that the rain replenishing the resources brings an end to the epidemic during the rainy season [14]. Mayotte experiences the same epidemic dynamic, with a surge of cases of diarrhoea in austral winter, a dry, temperate season from June to September.

The various surveillance networks in Mayotte show the burden of diarrhoea rising for several years, especially in children: in 2016, one in ten children under 15 (9.4%) were diagnosed with acute gastroenteritis in an emergency department, and this figure has been on the rise since 2013 when it was around 3.9% [15]. It would have been interesting to collect the age of the patients to identify a subpopulation potentially more impacted by water restriction. With children representing more than half of Mayotte’s population [16], the phenomenon probably affected a large part of this particularly vulnerable population. This has already been demonstrated in England during the drought of 1976, where surveillance of episodes of diarrhoea and vomiting in the schools of two regions affected by water restrictions showed a link between the restrictions and gastrointestinal disorders [17]. Faced with such a vulnerable population (58% of the population are illiterate and unschooled [18]), sustained prevention campaigns are needed during epidemic or crisis period but also all year long, like those carried out following a diarrhoea epidemic in the South Pacific islands during a serious drought crisis associated with the La Niña phenomenon in 2011 [19].

Moreover, the threat of importing cholera cases still looms over Mayotte. Indeed, in the 1990s a cholera epidemic hit the Indian Ocean region from East Africa, affecting the Comoros islands, Madagascar and in 2000, Mayotte. Ten cases were identified at the time [20]. Since then, outbreaks of cholera have occurred regularly in southern Africa, the Horn of Africa, and the Gulf of Aden. Outbreaks have also been observed in West Africa more recently [21]. Given the epidemiological situation of cholera in East Africa, Mayotte’s geographic proximity with these countries, population movements and the precarious living conditions of part of Mayotte’s population, the risk of importing and spreading the disease remains pertinent and vigilance is essential. This situation should lead to a reflection on alternative techniques for conserving water resources. Restrictions on access to water could contribute to the spread of such oral-fecal diseases.

About skin diseases, although the surveillance system revealed no significant difference between the island’s different communes, it did highlight the undeniable burden of infections like abscesses, fungal infections and impetigo in Mayotte. These pathologies are stimulated by heat, humidity, maceration, perspiration and a lack of hygiene. Besides, many health signals were reported to the CIRE OI and the ARS OI during the crisis, especially from private practice nurses concerning the deterioration of the health status of patients hospitalised at home with no access to water. Many cases of infectious diseases affecting the skin or mucous membranes, and superinfection of dressings were described. In a context where there is a high prevalence of streptococcal and staphylococcal infections (data available from authors), increased vigilance is needed for patients with skin lesions to avoid relapses or the spread of this bacteria into the community. In April 2017, skin diseases were therefore added to the indicators to be monitored routinely by Mayotte’s network of sentinel doctors. It seemed relevant to monitor the spatial and temporal changes in these diseases so that this problem can be clearly identified as a real public health issue in Mayotte and to alert public health authorities for action.

A particular phenomenon was observed over the leptospirosis epidemic period which the water crisis overlapped. From December to March, a higher number of cases of leptospirosis were reported in the areas affected by the water scarcity, whereas historically, the north of the island has been more prone to rainfall causing more cases in that part of the island. Analysis of the geographical distribution of cases over the last four years indicates a possible change in the epidemiology of leptospirosis in Mayotte. It could be possible that this change is due to global climate changes that are changing the rainfall’s frequency and intensity. Moreover, in villages deprived of water in 2017, the inhabitants used surface water for washing, food and hygiene, which could have been contaminated by rodents, domestic or farm animals carrying leptospira, promoting the onset of cases in the villages.

All the available monitoring networks were used during the water crisis. Data from the sentinel pharmacist network were essential to highlight the deterioration of the health status of the population. In this regard, the analysis of drug consumption seems to have been an indicator of very good quality as it involved the same extraction method for all pharmacists with a minimal risk of error (quickly identified during data processing). There were no variation from one pharmacy to another as opposed to doctors who tend to treat each clinical case differently. Besides, the network of sentinel doctors experienced more limitations: first of all, the fluctuating participation rate (because of vacations, departure from the territory, etc.), but also the participation to the network that is being based on volunteering. Also, sentinel doctors are unequally set on the island (two doctors are based in the center/south area against nine in the north). Finally, comparisons with reliable historical data for the center/south area were only possible for the years 2014/2015 (few data for 2015/2016). These comparisons were still very important information as the specific epidemiological monitoring showed a significant result only after a one-month delay. That’s why, they hardly convinced authorities of the occurrence of one unusual health situation in real time. As a consequence, the lightening of the restrictive measure was only put in place after two months. This demonstrates the value of having a responsive and sustainable surveillance system with targeted indicators based on local epidemiology, to help public authorities make decisions.

The specific epidemiological monitoring made it possible to highlight a significant difference between the two areas studied but it required extremely time-consuming organisation. The networking (recruitment, information, awareness and organisation) was a big workload. Weekly visits to the CRs were essential to maintain a relationship of trust with data providers and ensure continuity in reporting and better data quality. Although it would have been interesting to monitor many syndromes (respiratory, oto-laryngeal...), the collection could not be too ambitious and had to be focused on the most relevant indicators, given the context and the local epidemiology. Indeed, in times of crisis, CRs are overloaded and clinicians struggle to dedicate time for reporting. Besides, data were reported on paper which made it time consuming for the team of the CIRE OI to enter them. An online form, via smartphone for example, would have been more appropriate and probably quickly taken in hand by health professionals. This kind of tool could therefore be developed upstream for implementation in crisis situations.

Overall, it’s likely that the situation has been underestimated across all surveillance networks. Indeed, in Mayotte, patients do not systematically consult for diarrhoea symptomatology and even less for a cutaneous symptomatology. According to hospital clinicians, patients are often hospitalized at very advanced stages of their pathology because of the important problem of access to care in Mayotte.

Following this crisis, many questions arose in terms of surveillance and health alert. A working group on the water-borne/oral-fecal diseases brought together the CIRE OI and the various services of the ARS OI (health-environment, vector control, alert and health management). The objective of this group is to develop recommendations about waterborne diseases in Mayotte and propose a plan of action through the reorganisation of the investigations around clusters of acute diarrhoea, gastro-enteritis, typhoid, hepatitis A; and provide support for prevention and health promotion actions. In this same dynamic, the CIRE OI is currently working on a community-based surveillance system whose objective is to integrate a health approach involving community members in the identification and notification of epidemic-prone disease. This surveillance, as close as possible to the field and the most vulnerable populations (often far removed from the healthcare system), would be complementary to surveillance networks developed with health professionals.

## Conclusion

Water scarcity can occur at any time, adding to the already extremely fragile water resource situation in Mayotte. It is a real risk factor for the deterioration of the population’s health status. Therefore, long-term close monitoring of the epidemiological situation across the territory in collaboration with all local partners is crucial. The existing surveillance system thus needs to be adapted so it can more effectively monitor the changing health status of Mayotte’s ever-growing population, with 256,500 inhabitants counted during the last census in 2017 [22]. Since January 2017, the CIRE OI has regularly strengthened its network of sentinel doctors within the CRs and in the limited number of private doctors. “Thanks to the water crises”, the CIRE OI met new interlocutors and considerably expanded its network of partners. Today, twenty-five doctors spread across the territory report the weekly number of flu-like syndromes, cases of acute diarrhoea, dengue-like syndromes, bronchiolitis and skin diseases. These data are collated and compared with data from the OSCOUR® network (monitoring of hospital emergency passages) analysed daily. Lastly, surveillance of leptospirosis, arboviruses and malaria is being maintained and continually strengthened.

It is clear that the prevalence of pathologies linked to poor-quality water in Mayotte could be dramatically reduced if large-scale measures were taken. Indeed, alongside close epidemiological monitoring, it seems essential to adopt a holistic approach, both from a structural perspective, by giving all inhabitants access to water and sanitation, and in terms of health promotion and disease prevention.

## Abbreviations

ARS OI: Agence de santé ocean Indien [Indian Ocean Health Agency]
CHM: Centre Hospitalier de Mayotte [Mayotte Hospital Center]
CIRE OI: cellule d’intervention en region ocean Indien [French public health agency in the Indian Ocean region]
CR: centre de référence du Centre Hospitalier de Mayotte [CHM’s referral center]
INSEE: Institut national de la statistique et des études économiques [National Institute of Statistics and Economic studies]
ORS: oral rehydration solution
OSCOUR: Organisation de la Surveillance COrdonnée des Urgences [Network for monitoring of hospital emergency]
W06–2017: week 6 of 2017
